# An integrated machine learning predictive scheme for longitudinal laboratory data to evaluate the factors determining renal function changes in patients with different chronic kidney disease stages

**DOI:** 10.3389/fmed.2023.1155426

**Published:** 2023-10-04

**Authors:** Ming-Hsien Tsai, Mao-Jhen Jhou, Tzu-Chi Liu, Yu-Wei Fang, Chi-Jie Lu

**Affiliations:** ^1^Division of Nephrology, Department of Medicine, Shin Kong Wu Ho-Su Memorial Hospital, Taipei, Taiwan; ^2^Department of Medicine, School of Medicine, Fu Jen Catholic University, New Taipei City, Taiwan; ^3^Graduate Institute of Business Administration, Fu Jen Catholic University, New Taipei City, Taiwan; ^4^Artificial Intelligence Development Center, Fu Jen Catholic University, New Taipei City, Taiwan; ^5^Department of Information Management, Fu Jen Catholic University, New Taipei City, Taiwan

**Keywords:** chronic kidney disease, diabetes, machine learning, estimated glomerular filtration, feature selection

## Abstract

**Background and objectives:**

Chronic kidney disease (CKD) is a global health concern. This study aims to identify key factors associated with renal function changes using the proposed machine learning and important variable selection (ML&IVS) scheme on longitudinal laboratory data. The goal is to predict changes in the estimated glomerular filtration rate (eGFR) in a cohort of patients with CKD stages 3–5.

**Design:**

A retrospective cohort study.

**Setting and participants:**

A total of 710 outpatients who presented with stable nondialysis-dependent CKD stages 3–5 at the Shin-Kong Wu Ho-Su Memorial Hospital Medical Center from 2016 to 2021.

**Methods:**

This study analyzed trimonthly laboratory data including 47 indicators. The proposed scheme used stochastic gradient boosting, multivariate adaptive regression splines, random forest, eXtreme gradient boosting, and light gradient boosting machine algorithms to evaluate the important factors for predicting the results of the fourth eGFR examination, especially in patients with CKD stage 3 and those with CKD stages 4–5, with or without diabetes mellitus (DM).

**Main outcome measurement:**

Subsequent eGFR level after three consecutive laboratory data assessments.

**Results:**

Our ML&IVS scheme demonstrated superior predictive capabilities and identified significant factors contributing to renal function changes in various CKD groups. The latest levels of eGFR, blood urea nitrogen (BUN), proteinuria, sodium, and systolic blood pressure as well as mean levels of eGFR, BUN, proteinuria, and triglyceride were the top 10 significantly important factors for predicting the subsequent eGFR level in patients with CKD stages 3–5. In individuals with DM, the latest levels of BUN and proteinuria, mean levels of phosphate and proteinuria, and variations in diastolic blood pressure levels emerged as important factors for predicting the decline of renal function. In individuals without DM, all phosphate patterns and latest albumin levels were found to be key factors in the advanced CKD group. Moreover, proteinuria was identified as an important factor in the CKD stage 3 group without DM and CKD stages 4–5 group with DM.

**Conclusion:**

The proposed scheme highlighted factors associated with renal function changes in different CKD conditions, offering valuable insights to physicians for raising awareness about renal function changes.

## 1. Introduction

Chronic kidney disease (CKD), characterized by decreased glomerular filtration rate, is a global health concern with a high prevalence rate (10–15%); moreover, this disease is highly associated with morbidity and mortality, leading to financial and medical burdens (1–3). The gradual loss of kidney function in patients with CKD may lead to end-stage kidney disease (ESKD) that requires kidney replacement therapy. According to the latest annual report of the United States Renal Data System, the average annual increase in the ESKD prevalence worldwide from 2003 to 2016 ranged from 0.1 to 109 per million population (4), thus placing a greater burden on the health insurance system of many countries. The ESKD prevalence in Taiwan is high (4), with a substantial increase observed between 2010 and 2018 (5).

Timely intervention for delaying the progression of CKD to ESKD may not only improve the quality of life of patients but also reduce the associated morbidity and mortality (6, 7). As the exacerbation of renal function in patients with CKD is usually silent, it is clinically important to develop an accurate prediction model for the risk of CKD progression. Such a model is expected to facilitate physicians in making personalized treatment decisions, thereby improving the overall prognosis. Various statistical models have been developed to predict the risk of ESKD based on variables such as age, sex, blood pressure, comorbidities, laboratory data, and most commonly, the estimated glomerular filtration rate (eGFR) and proteinuria level (8). Among them, the most popular statistical model is the four-variable kidney failure risk equation (KFRE) based on age, sex, eGFR, and urine albumin-to-creatine ratio (9). Further, an eight-variable equation based on KFRE (which further included serum albumin, bicarbonate, calcium, and phosphate levels) was proposed to provide a more accurate prediction (10). The traditional statistical methods are often based on predefined hypotheses and assumptions. Researchers formulate hypotheses and test them using statistical methods. Moreover, these methods often focus on drawing inferences and conclusions about a population based on a sample. These methods aim to provide insights into causal relationships and generalizability (11). However, the traditional statistical methods have several limitations in effectively dealing with the challenges posed by big data and complex data structures.

Machine learning (ML) methods excel in analyzing unstructured data and complex patterns, whereas traditional statistical methods often require human intervention and expertise in model selection, hypothesis formulation, and result interpretation (11). In the big data era, several applications of ML, which is a subset of artificial intelligence (AI), have emerged in health informatics (12), allowing computers to perform a specific task without direct instruction (13). In contrast to theory-driven formula that requires a predefined hypothesis based on prior knowledge, ML models typically follow a data-driven approach that allows the model to learn from experience alone (14). Therefore, compared with the traditional statistical methods, ML models may demonstrate better performance in predicting a determined outcome, as they have no strict assumptions when modeling (15–21). The utilization of ML algorithms in CKD is a promising research topic that aims at assisting healthcare professionals in diagnosing and managing patients with CKD via computer-aided decision support systems for the early identification of critical events such as ESKD or eGFR doubling (22–25).

To date, only a few studies have used ML methods in CKD populations for identifying metabolomic signatures of pediatric CKD etiology (26), developing a lifestyle scoring system for CKD prevention (27), using retinal images and clinical metadata to predict eGFR and CKD stage (28), and predicting incident ESKD in patients with diabetes mellitus (DM) and CKD (29). However, previous ML prediction models have primarily integrated baseline laboratory data and clinical information, and many studies have focused on predicting ESKD rather than eGFR changes (25). Furthermore, studies on CKD-related risk factor screening or analysis have predominantly relied on a single model without considering hybrid approaches, especially when employing ML methods (26, 27, 29, 30). According to a previous study, the mean annual eGFR decline in healthy individuals was estimated to be 0.97 ± 0.02 mL/min/1.73 m^2^ (31). However, even for individuals with the same underlying comorbidities or extent of kidney function impairment, the eGFR decline could be highly variable. Early identification and management of patients with CKD based on longitudinal biochemical data are essential.

Therefore, this study aimed to identify significant factors that influence the prediction of eGFR changes using the ML and important variable selection (ML&IVS) scheme in a CKD cohort with longitudinal laboratory data. In the context of longitudinal data analysis, it is crucial to prioritize the examination of the rate and variation of biochemical data rather than solely relying on baseline data. This study employed five effective ML algorithms, namely, stochastic gradient boosting (SGB), multivariate adaptive regression splines (MARS), random forest (RF), eXtreme gradient boosting (XGBoost), and light gradient boosting machine (LightGBM). Using these algorithms, we developed an integrated multistage ML-based predictive scheme for all four subgroups according to eGFR and presence of DM to predict eGFR changes and subsequently evaluate and integrate relatively important risk factors.

## 2. Materials and methods

### 2.1. Dataset

This retrospective study included 710 patients with nondialysis-dependent CKD who were recruited from outpatient nephrology clinics of the Shin-Kong Wu Ho-Su Memorial Hospital Medical Center for a prospective cohort study from 2016 to 2021. The inclusion criteria were as follows: patients aged ≥20 years, those who sustained (≥3 months) a decrease in eGFR of ≤60 mL/min/1.73 m^2^ based on the four-variable modification of diet in renal disease study equation (32), and those who were regularly followed up in our CKD multidisciplinary care program (33). Patients who did not visit the nephrological outpatient department for ≥4 months and had incomplete data were excluded. In our CKD multidisciplinary care program, patients were regularly followed up in the nephrological outpatient department every 3 months.

This study was conducted according to the guidelines of the Declaration of Helsinki and was approved by the Institutional Review Board of the Shin-Kong Wu Ho-Su Memorial Hospital, Taipei, Taiwan (IRB no. 20200901R). Informed consent was waived because our study was based on a medical chart review. Patient information was anonymized and de-identified before analysis.

### 2.2. Definition of longitudinal variables

This study aimed to predict eGFR changes and the corresponding relationship between risk factors in the fourth examination of each patient; thus, the results of the first three examinations of each patient were utilized as independent variables. As the results of the first three examinations were collected from each patient, the independent variables used in this study could be regarded as longitudinal predictor variables.

The definitions of the longitudinal variables used in this study are presented in Table 1. As shown, *V*_*i,t*_ represents the *t*_*th*_ time examination result of *i*_*th*_ variable [e.g., *V*_1,2_ is the systolic blood pressure (SBP) result of the second examination]. A total of 17 variables were utilized in this study. Moreover, as all patients had previous records of their first three examinations, the variables used in this study could be further defined as Equation (1).


(1) $$V_{i,t},\forall i,t\in \mathbb{N}$$

where 1 ≤ *i* ≤ 17; 1 ≤ *t* ≤ 3.

**TABLE 1:** Definition of longitudinal variables.

	Variables	Description
*V*_1,*t*_	SBP	SBP in the *t*_*th*_ examination
*V*_2,*t*_	DBP	DBP in the *t*_*th*_ examination
*V*_3,*t*_	Height	Height in the *t*_*th*_ examination
*V*_4,*t*_	Weight	Weight in the *t*_*th*_ examination
*V*_5,*t*_	HB	HB in the *t*_*th*_ examination
*V*_6,*t*_	BUN	BUN in the *t*_*th*_ examination
*V*_7,*t*_	UA	UA in the *t*_*th*_ examination
*V*_8,*t*_	Na	Na in the *t*_*th*_ examination
*V*_9,*t*_	K	K in the *t*_*th*_ examination
*V*_10,*t*_	iCa	iCa in the *t*_*th*_ examination
*V*_11,*t*_	P	P in the *t*_*th*_ examination
*V*_12,*t*_	Albumin	Albumin in the *t*_*th*_ examination
*V*_13,*t*_	TG	TG in the *t*_*th*_ examination
*V*_14,*t*_	LDL	LDL in the *t*_*th*_ examination
*V*_15,*t*_	AC Sugar	AC sugar in the *t*_*th*_ examination
*V*_16,*t*_	UP	UP in the *t*_*th*_ examination
*V*_17,*t*_	eGFR	eGFR value in the *t*_*th*_ examination
*Y*	eGFR in the fourth examination	eGFR value in the fourth examination

AC, before meals; BUN, blood urea nitrogen; DBP, diastolic blood pressure; eGFR, estimated glomerular filtration rate; Hb, hemoglobin; iCa, ionized calcium; LDL, low-density lipoprotein; P, phosphate; SBP, systolic blood pressure; TG, triglycerides; UA, uric acid; UP, urine protein.

Then, this study utilized three approaches for generating more predictor variables that can be used to construct the ML predictive models. These three approaches, namely, “closest,” “mean,” and “standard deviation,” could provide various data from the independent variables. The “closest” approach generated the predictor variable using the result of the latest examination, which is the third examination result (*V*_*i*,3_) in this study. The predictor variable generated using the “closest” approach (*V_i_C*) was defined as Equation (2). For example, *V*_1_*C* is the SBP result of the third examination (*V*_1,3_) and can be written as SBP(C).

The “mean” approach generated the predictor variable by calculating the mean of the results of the first three examinations (*V*_*i*,1_, *V*_*i*,2_, *V*_*i*,3_). The predictor variable generated using the “mean” approach (*V_i_M*) was defined as Equation (3). For demonstration, *V*_1_*M* was constructed by calculating the mean of the results of the first (*V*_1,1_), second (*V*_1,2_), and third (*V*_1,3_) SBP examinations. *V*_1_*M* can also be written as SBP(M).

Similar to the concept of the “mean” approach, the “standard deviation” approach generated the predictor variable (*V_i_S*) by calculating the standard deviation of the results of the first three examinations (*V*_*i*,1_, *V*_*i*,2_, *V*_*i*,3_), as shown in Equation (4). For example, *V*_1_*S* is the standard deviation of the results of the first three SBP examinations (*V*_1,1_, *V*_1,2_, *V*_1,3_) and can be written as SBP(S).


(2) $$V_{i}\,C=V_{i,3}$$

(3) $$V_{i}\,M=M\,e\,a\,n\,\left(V_{i,1},V_{i,2},V_{i,3}\right)$$

(4) $$V_{i}\,S=S\,t\,d\,\left(V_{i,1},V_{i,2},V_{i,3}\right)$$

The above mentioned 3 approaches were utilized for all 17 independent variables to generate the overall predictor variables used in this study. In addition, as height and weight do not change drastically between examinations, this study only used the “closest” approach for these variables. Thus, 47 predictor variables were generated and utilized for constructing the ML predictive models. The descriptive statistical data of 47 predictor variables and the target variable (eGFR of the fourth examination) are presented in Table 2.

**TABLE 2:** Descriptive statistical data of 47 predictor variables and the target variable.

Predictor variables	Abb. Name	*V*_*i*_*C* (Mean ± SD)	*V*_*i*_*M* (Mean ± SD)	*V*_*i*_*S* (Mean ± SD)
V_1_	SBP (mmHg)	133.46 ± 16.1	134.93 ± 14.25	10.43 ± 7.65
V_2_	DBP (mmHg)	71.26 ± 9.98	71.88 ± 8.64	7.18 ± 5.36
V_3_	Height (cm)	159.99 ± 8.99	–	–
V_4_	Weight (kg)	67.63 ± 13.29	–	–
V_5_	Hb (g/dL)	11.88 ± 2.02	11.91 ± 1.93	0.58 ± 0.47
V_6_	BUN (mg/dL)	36.12 ± 17.9	36.01 ± 15.41	6.18 ± 5.62
V_7_	UA (mg/dL)	5.91 ± 1.86	6.18 ± 1.46	1.23 ± 1.07
V_8_	Na (MEq/L)	140 ± 3.13	139.83 ± 2.63	1.66 ± 1.38
V_9_	K (MEq/L)	4.51 ± 0.54	4.52 ± 0.47	0.3 ± 0.21
V_10_	iCa (mg/dL)	4.67 ± 0.27	4.67 ± 0.22	0.15 ± 0.11
V_11_	P (mg/dL)	3.78 ± 0.72	3.79 ± 0.61	0.36 ± 0.26
V_12_	Albumin (g/dL)	4.08 ± 0.4	4.07 ± 0.38	0.16 ± 0.12
V_13_	TG (mg/dL)	156.87 ± 105.69	162.04 ± 101.39	42.34 ± 66.47
V_14_	LDL (mg/dL)	85.99 ± 30.04	88.1 ± 24.2	15.7 ± 14.71
V_15_	AC sugar (mg/dL)	113.34 ± 37.74	114.63 ± 32.29	16.92 ± 22.7
V_16_	UPCR (mg/mg)	1,192.38 ± 1,936.41	1,236.66 ± 1,980.14	415.84 ± 732.27
V_17_	eGFR (mL/min/1.73 m^2^)	31.93 ± 12.94	31.85 ± 11.65	3.49 ± 3.05
**Target variable (*Y*)**		***N***		**Mean ± SD**
eGFR in the fourth examination		710		31.59 ± 13.29

*V_i_C*, closest value; *V_i_M*, mean value; *V_i_S*, standard deviation; AC, before meals; BUN, blood urea nitrogen; DBP: diastolic blood pressure; eGFR, estimated glomerular filtration rate; Hb, hemoglobin; iCa, ionized calcium; LDL, low-density lipoprotein; P, phosphate; SBP, systolic blood pressure; TG, triglycerides; UA, uric acid; UPCR, urine protein-to-creatinine ratio.

### 2.3. Proposed scheme

**FIGURE 1:**
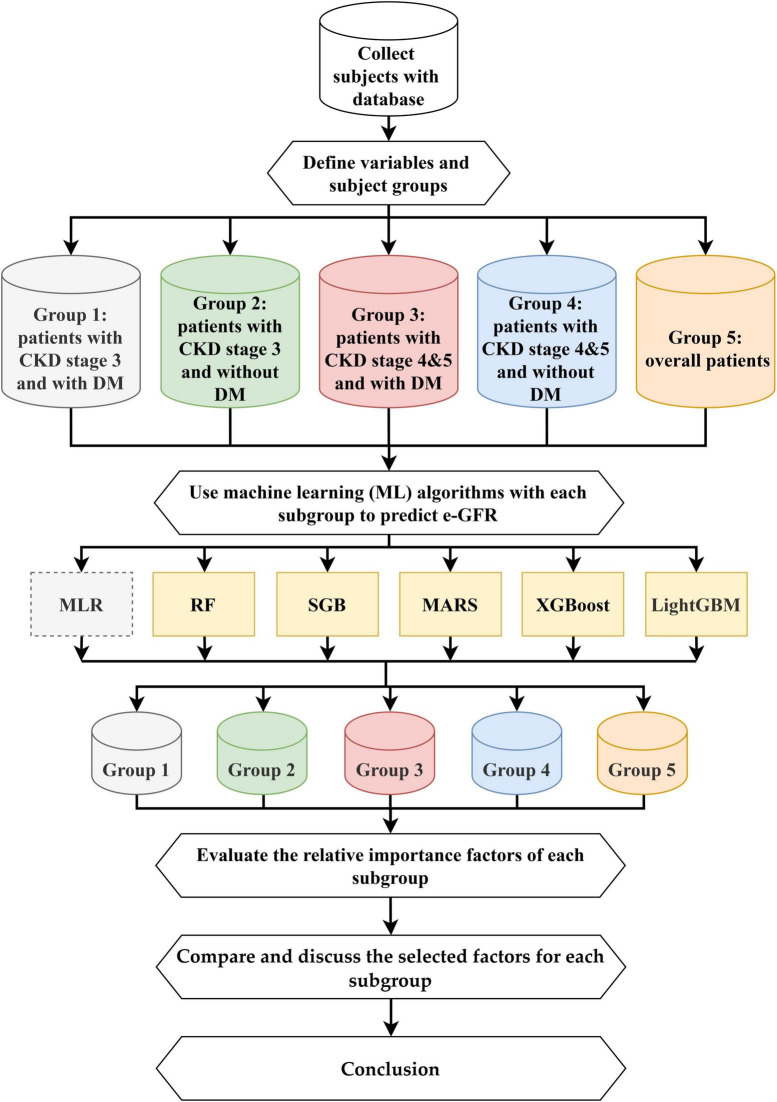
Proposed machine learning predictive and important variable selection scheme (ML&IVS).

This study proposed an integrated multistage predictive scheme known as ML&IVS based on five ML methods, including SGB, MARS, RF, XGBoost, and LightGBM, for predicting eGFR changes and subsequently identifying important risk factors. The five ML algorithms have been successfully used in various medical/healthcare applications (16, 19, 21, 23, 24, 34–37). The overall process of ML&IVS scheme is shown in Figure 1.

In this process, first, 4-year health examination data of patients with CKD were collected; the dataset is described in Section “2.1. Dataset.” Second, 47 longitudinal risk variables were defined, and the patients were categorized into five subgroups: Group 1 (patients with CKD stage 3 and DM), Group 2 (patients with CKD stage 3 without DM), Group 3 (patients with CKD stages 4–5 and DM), Group 4 (patients with CKD stages 4 and 5 without DM), and Group 5 (overall patients). The definitions of variables are presented in Section “2.2. Definition of longitudinal variables.”

Third, RF, SGB, MARS, XGBoost, and LightGBM were used to construct predictive models for all five groups. As multiple linear regression (MLR) is a commonly used statistical method in medical/healthcare applications, it was used in this study as a bench method. RF is an ensemble learning decision tree algorithm that combines bootstrap resampling and bagging (38). The RF principle entails random generation of many different and unpruned classification and regression tree (CART) decision trees, in which the decrease in Gini impurity is regarded as the splitting criterion, and all generated trees are combined into a forest. Then, all trees in the forest are averaged or voted to generate output probabilities and construct the final robust model. SGB is a tree-based gradient boosting learning algorithm that combines both bagging and boosting techniques to minimize the loss function and thereby solve the overfitting problem of the traditional decision trees (39, 40). SGB comprises many stochastic weak learners of decision trees that are sequentially generated through multiple iterations, in which each tree focuses on correcting or explaining the errors of the tree generated in the previous iteration. In other words, the residual of the previous iteration tree is used as the input for the newly generated tree. This iterative process is repeated until a convergence condition or stopping criterion is reached for the maximum number of iterations. Finally, the cumulative results of several trees are used to construct the final robust model. MARS is a nonlinear spline regression method and nonparametric form of the regression analysis algorithm (41). MARS uses multiple piecewise linear segments (splines) with different gradients. It considers each sample as a knot and divides it into several sections for the successive linear regression analysis of data within each section. For determining knots, a forward algorithm is used to select all possible basic functions and their corresponding knots, and a backward algorithm is used to eliminate all basic functions to generate the best combinations of existing knots.

XGBoost is a gradient boosting technology based on an SGB-optimized extension (42). It trains many weak models sequentially to ensemble them using the gradient boosting method of outputs, which can achieve a promising prediction performance. In XGBoost, Taylor binomial expansion is used to approximate the objective function and arbitrary differentiable loss functions, thereby accelerating the convergence process of model construction. Then, XGBoost employs a regularized boosting technique to penalize the complexity of the model and correct overfitting, thus increasing the accuracy of the model. LightGBM is a decision tree-based distributed gradient boosting framework that uses the decision tree technique with improved histograms. To improve the accuracy of tree models, LightGBM uses gradient-based one-sided sampling and a unique feature bundling algorithm to fit a loss function with negative gradients and learn the residual approximation of decision trees in iterations (43).

Fourth, after constructing effective ML models for all five groups, the relative importance values of each risk factor can be obtained via ML algorithms. In this study, the most important risk factor had an importance value of 100, whereas the least important risk factor had an importance value of 0. Values can be repeated, i.e., two or more variables can have similar variable importance. Because different ML algorithms have different calculus architectures and selection features, the five ML algorithms in this study may generate different variable importance values for a single risk factor. Within the same group, a single, robust, and complete variable importance value can be generated for each risk factor to facilitate subsequent comparison of variable rankings and identification of important risk factors. A single pooled value of variable importance was generated based on the average value of variable importance derived from the five ML models.

Fifth, the important variables among groups were compared, and their differences were discussed. Sixth, the conclusions of this study were presented based on the abovementioned findings.

For constructing each model, nested cross-validation (Nest-CV) was utilized. Nest-CV is a variation of the CV family. Under the structure of Nest-CV, two loops are required (inner and outer loops). The inner loop is used for hyperparameter tuning (which is equal to k-fold CV), whereas the outer loop is used for model evaluation, with the optimal hyperparameter set in the inner loop. Nest-CV is commonly utilized, and several studies have demonstrated that Nest-CV can overcome the problem of overfitting effectively (44–47).

During model construction under Nest-CV structure, the outer loop first randomly splits the dataset into several folds (10 folds in this study). Then, for each iteration, one fold of the data from the outer loop is used for testing, whereas the remaining folds are used for training. Next, the training data from the inner loop are used for hyperparameter tuning with 10-fold CV. In the 10-fold CV approach, the training dataset was further randomly divided into 10 folds, wherein 9 folds were used to construct the model with a different set of hyperparameters, and 1 fold was used for validation. All 10 folds were used for validation at least once, and all possible combinations of the hyperparameters were investigated using a grid search. The hyperparameter set during validation with the lowest root mean square error (RMSE) was regarded as the most optimized set. After determining the set and training the model using the set, the test data from the outer loop were used for evaluation, which involves the completion of one iteration. Entire Nest-CV was completed when each fold of the data from the outer loop was used for testing at least once. After constructing the ML models, the variable importance values can be extracted from these models. As 10-fold Nest-CV is utilized, the extracted variable importance ranking from each ML model will have 10 scores for each variable. Therefore, to obtain the final corresponding variable importance values, the approach of averaging the importance scores for each variable was employed.

As the target variable of this study (changes in eGFR) for model construction is a numerical variable, the metrics used for model performance comparison included symmetric mean absolute percentage error (SMAPE), mean absolute percentage error (MAPE), relative absolute error (RAE), root relative squared error (RRSE), and RMSE (Table 3). In addition, “R” software version 3.6.2 and “RStudio” version 1.1.453 were used for model construction. The related “R” packages utilized for constructing RF, SGB, MARS, XGBoost, and LightGBM included “randomForest” package version 4.7-1.1 (48), “gbm” package version 2.1.8 (49), “earth” package version 5.3.1 (50), “XGBoost” package version 1.5.0.2 (51), and “lightgbm” package version 3.3.2, respectively (52). The “caret” package version 6.0-92 (53) was used in all algorithms to estimate the optimal hyperparameters for constructing the best prediction model.

**TABLE 3:** Equations of performance metrics.

Metrics	Description	Calculation
SMAPE	Symmetric mean absolute percentage error	$S\,M\,A\,P\,E=\frac{1}{n}\,\sum_{i=1}^{n}\frac{\left|y_{i}-{\hat{y}}_{i}\right|}{\left(\left|y_{i}\right|+\left|{\hat{y}}_{i}\right|\right)/2}\times 100$
MAPE	Mean absolute percentage error	$M\,A\,P\,E=\frac{1}{n}\,\sum_{i=1}^{n}\left|\frac{y_{i}-{\hat{y}}_{i}}{y_{i}}\right|\times 100$
RAE	Relative absolute error	$R\,A\,E=\sqrt{\frac{\sum_{i=1}^{n}{\left(y_{i}-{\hat{y}}_{i}\right)}^{2}}{\sum_{i=1}^{n}{\left(y_{i}\right)}^{2}}}$
RRSE	Root relative squared error	$R\,R\,S\,E=\sqrt{\frac{\sum_{i=1}^{n}{\left(y_{i}-{\hat{y}}_{i}\right)}^{2}}{\sum_{i=1}^{n}{\left(y_{i}-{\hat{y}}_{i}\right)}^{2}}}$
RMSE	Root mean squared error	$R\,M\,S\,E=\sqrt{\frac{1}{n}\,\sum_{i=1}^{n}{\left(y_{i}-{\hat{y}}_{i}\right)}^{2}}$

where ${\hat{y}}_{i}$ and $y_{i}$ represent predicted and actual values, respectively; *n*, number of instances.

## 3. Empirical study

The dataset was divided into five subgroups, i.e., Group 1 (patients with CKD stage 3 and DM), Group 2 (patients with CKD stage 3 without DM), Group 3 (patients with CKD stages 4–5 and DM), Group 4 (patients with CKD stages 4 and 5 without DM), and Group 5 (overall patients). All groups followed the same modeling process for predicting eGFR in the fourth examination. The performance of the methods used in all five groups is presented in Table 4.

**TABLE 4:** Performance of the MLR and five ML methods in all five groups.

Subgroup, total *N* = 710	Methods	SMAPE	MAPE	RAE	RRSE	RMSE
Group 1 (patients with CKD stage 3 and DM) *n* = 200 (28.17%)	MLR	0.116 (0.01)	0.118 (0.01)	0.799 (0.08)	0.821 (0.09)	5.886 (0.71)
	RF	0.098 (0.01)	0.099 (0.01)	0.680 (0.06)	**0.688 (0.05)**	4.926 (0.37)
	SGB	**0.097 (0.01)**	**0.098 (0.01)**	**0.672 (0.07)**	0.688 (0.06)	**4.922 (0.35)**
	MARS	0.099 (0.01)	0.100 (0.01)	0.687 (0.09)	0.715 (0.07)	5.121 (0.38)
	XGBoost	0.099 (0.01)	0.100 (0.01)	0.691 (0.07)	0.708 (0.05)	5.066 (0.33)
	LightGBM	0.099 (0.01)	0.100 (0.01)	0.687 (0.06)	0.702 (0.05)	5.028 (0.39)
Group 2 (patients with CKD stage 3 without DM) *n* = 200 (28.17%)	MLR	0.126 (0.02)	0.126 (0.02)	0.816 (0.13)	0.831 (0.11)	6.132 (0.52)
	RF	0.103 (0.01)	0.105 (0.01)	0.686 (0.08)	0.717 (0.08)	5.294 (0.35)
	SGB	**0.102 (0.01)**	**0.104 (0.01)**	**0.682 (0.09)**	**0.713 (0.09)**	**5.259 (0.45)**
	MARS	0.109 (0.01)	0.112 (0.01)	0.724 (0.10)	0.768 (0.09)	5.676 (0.40)
	XGBoost	0.106 (0.01)	0.107 (0.01)	0.707 (0.09)	0.741 (0.09)	5.478 (0.44)
	LightGBM	0.110 (0.01)	0.112 (0.01)	0.730 (0.07)	0.740 (0.08)	5.463 (0.28)
Group 3 (patients with CKD stages 4–5 and DM) *n* = 185 (26.06%)	MLR	0.217 (0.03)	0.238 (0.05)	0.625 (0.05)	0.694 (0.03)	4.838 (0.35)
	RF	**0.135 (0.03)**	**0.147 (0.04)**	**0.383 (0.07)**	**0.432 (0.08)**	**3.009 (0.62)**
	SGB	0.141 (0.03)	0.155 (0.04)	0.397 (0.06)	0.438 (0.08)	3.056 (0.60)
	MARS	0.145 (0.02)	0.152 (0.03)	0.433 (0.09)	0.507 (0.11)	3.519 (0.70)
	XGBoost	0.147 (0.02)	0.161 (0.03)	0.411 (0.05)	0.457 (0.07)	3.191 (0.55)
	LightGBM	0.146 (0.03)	0.163 (0.04)	0.407 (0.06)	0.448 (0.08)	3.124 (0.62)
Group 4 (patients with CKD stages 4–5 without DM) *n* = 125 (17.61%)	MLR	0.280 (0.04)	0.296 (0.05)	0.634 (0.10)	0.707 (0.09)	5.068 (0.43)
	RF	**0.159 (0.02)**	**0.178 (0.03)**	**0.406 (0.05)**	**0.464 (0.07)**	**3.333 (0.51)**
	SGB	0.173 (0.03)	0.200 (0.03)	0.427 (0.07)	0.484 (0.08)	3.472 (0.57)
	MARS	0.197 (0.04)	0.200 (0.03)	0.482 (0.08)	0.557 (0.08)	3.997 (0.57)
	XGBoost	0.171 (0.03)	0.193 (0.04)	0.435 (0.07)	0.483 (0.07)	3.483 (0.59)
	LightGBM	0.175 (0.03)	0.206 (0.05)	0.436 (0.07)	0.486 (0.08)	3.495 (0.59)
Group 5 (overall patients) *n* = 710 (100%)	MLR	0.149 (0.01)	0.156 (0.01)	0.369 (0.04)	0.414 (0.04)	5.507 (0.28)
	RF	**0.138 (0.01)**	0.150 (0.01)	0.354 (0.04)	0.403 (0.04)	5.354 (0.33)
	SGB	0.145 (0.01)	0.160 (0.01)	0.362 (0.04)	0.406 (0.04)	5.397 (0.29)
	MARS	0.139 (0.01)	**0.147 (0.01)**	**0.350 (0.04)**	**0.397 (0.03)**	**5.281 (0.27)**
	XGBoost	0.144 (0.01)	0.157 (0.01)	0.361 (0.04)	0.405 (0.03)	5.394 (0.26)
	LightGBM	0.143 (0.01)	0.157 (0.01)	0.366 (0.04)	0.411 (0.04)	5.463 (0.28)

Bold values indicates the best performance in the subgroup.

As shown in Table 4, the error of the metrics and their corresponding standard deviation (SD) are presented; percentage values after *n* in the first column indicate the corresponding proportion of the group. In Group 1, ML methods showed better performance than the MLR method. Among these ML methods, SGB showed the best performance with the following values: SMAPE, 0.097; MAPE, 0.098; RAE, 0.672; and RMSE, 4.922. RF had similar RRSE as SGB but lower SD than SGB.

Similarly, in Group 2, ML methods outperformed the MLR method. Furthermore, SGB showed the best performance in this subgroup with the following values: SMAPE, 0.102; MAPE, 0.104; RAE, 0.682; RRSE, 0.713; and RMSE, 5.259. In Group 3, RF showed the best performance with the following values: SMAPE, 0.135; MAPE, 0.147; RAE, 0.383; RRSE, 0.432; and RMSE, 3.009.

In Group 4, RF showed the best performance with the following values: SMAPE, 0.159; MAPE, 0.178; RAE, 0.406; RRSE, 0.464; and RMSE, 3.333. Finally, in Group 5, the CKD stage 3–5 dataset was considered the full dataset without subgrouping. ML methods showed better performance than the MLR method. Among them, MARS showed the best performance with the following values: MAPE, 0.147; RAE, 0.350; RRSE, 0.397; and RMSE, 5.281.

**FIGURE 2:**
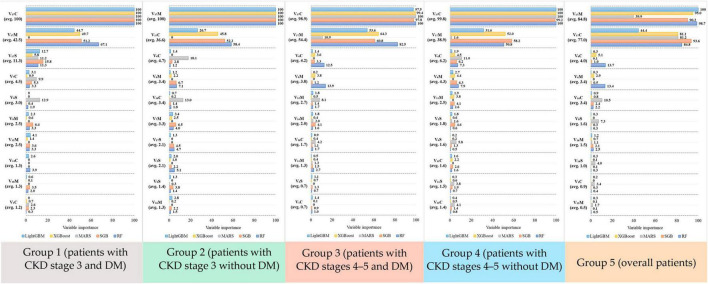
Variable importance score generated from the five algorithms for each risk factor in the five groups.

Overall, the performance of the five ML methods used in each subgroup had low SD, indicating that the ML usage in this study was reasonable and robust. Furthermore, different ML methods have different mechanisms for generating data regarding various risk factors, and each method visualizes data with a distinct perspective. In other words, ML methods can generate valuable information for supporting decision making with various perspectives; thus, the information generated from all ML methods can be considered. To accurately rank the risk factors using ML methods in different subgroups, this study revealed the top 10 risk factors in each subgroup (Figure 2).

As shown in Figure 2, in Group 1, risk factors were ranked by the five ML methods using the importance scores. Then, we calculated the average importance score of each risk factor to determine the top 10 important risk factors. Thus, in Group 1, the rank 1 risk factor was *V*_17_*C*, rank 2 was *V*_17_*M*, and rank 3 was *V*_17_*S*. Similarly, in Groups 2–5, the top 10 important risk factors were calculated. Further, to easily compare the important risk factors between subgroups, the results from Figure 2 were further displayed in Table 5 and the distribution plots of the top 10 important features of each subgroups are shown in Supplementary Figures 1–5.

**TABLE 5:** Ranking of the top 10 important variables of the five groups.

Subgroup	Group 1 (patients with CKD stage 3 and DM)	Group 2 (patients with CKD stage 3 without DM)	Group 3 (patients with CKD stages 4–5 and DM)	Group 4 (patients with CKD stages 4–5 without DM)	Group 5 (overall patients)
**Rank**	**Variable**	**Variable**	**Variable**	**Variable**	**Variable**
1	eGFR(C)	eGFR(M)	eGFR(C)	eGFR(C)	eGFR(M)
2	eGFR(M)	eGFR(C)	eGFR(M)	eGFR(M)	eGFR(C)
3	eGFR(S)	BUN(C)	BUN(C)	BUN(C)	BUN(C)
4	UA(C)	BUN(M)	BUN(M)	BUN(M)	BUN(M)
5	Na(S)	UPCR(C)	Albumin(M)	P(M)	UPCR(C)
6	Na(M)	UA(M)	UPCR(M)	Hb(S)	Na(C)
7	Albumin(M)	eGFR(S)	UPCR(C)	BUN(S)	UPCR(M)
8	Albumin(C)	Hb(S)	P(M)	P(C)	UPCR(S)
9	iCa(M)	K(S)	DBP(S)	P(S)	SBP(C)
10	SBP(C)	UPCR(M)	SBP(C)	Albumin(C)	TG(M)

eGFR(C): *V*_17_*C*; eGFR(M): *V*_17_*M*; eGFR(S): *V*_17_*S*; BUN(C): *V*_6_*C*; BUN(M): *V*_6_*M*; BUN(S): *V*_6_*S*; UA(C): *V*_7_*C*; UA(M): *V*_7_*M*; Na(C): *V*_8_*C*; Na(M): *V*_8_*M*; Na(S): *V*_8_*S*; UP(C): *V*_16_*C*; UP(M): *V*_16_*M*; UP(S): *V*_16_*S*; Albumin(C): *V*_12_*C*; Albumin(M): *V*_12_*M*; P(C): *V*_11_*C*; P(M): *V*_11_*M*; P(S): *V*_11_*S*; HB(S): *V*_5_*S*; iCa(M): *V*_10_*M*; K(S): *V*_9_*S*; SBP(C): *V*_1_*C*; SBP(S): *V*_1_*S*; DBP(S): *V*_2_*S*;TG(M): *V*_13_.

Table 5 presents the differences in the top 10 important risk factors among all 5 groups. eGFR(C) and eGFR (M) were the first (rank 1) and second (rank 2) important risk factors, respectively, in all groups. Interestingly, BUN(C) was the third important risk factor in all groups, except for Group 1. In general, the ranking of risk factors in each subgroup was different. For example, UA(C) only appeared in Group 1 and K(S) only appeared in Group 2.

## 4. Discussion

This study utilized longitudinal electronic health records to identify the risk factors associated with the prediction of eGFR changes in different CKD groups. This analysis considered the current values, mean values, and variation of biochemical data. Our proposed ML&IVS scheme yielded valuable results that can aid clinicians in effectively managing CKD progression and providing preventive measures in the CKD multidisciplinary care program. In addition to identifying important factors in CKD progression, our ML scheme, with some necessary modifications, can be seamlessly integrated into an electronic system, enabling the early identification of CKD progression. To the best of our knowledge, this study represents the first attempt to utilize ML methods to predict short-term eGFR changes in patients with CKD. The innovative application of ML in this context holds great potential for advancing the field and improving patient care.

ML has demonstrated promising performance in the nephrological field, including kidney function prediction via ultrasonography (54), acute kidney injury prediction in critical care (55, 56), specific pattern identification on renal pathology slides (57, 58), optimal dialysis prescription (59, 60), calculation of further eGFRs (61), mortality risk prediction in patients undergoing dialysis (62), and ESKD prediction based on clinical data (63–65). In this study, five ML methods were adopted to obtain the 10 most important factors for predicting eGFR changes in different CKD groups. The latest eGFR and mean eGFR were the two most important factors among all subgroups. Moreover, the SD of eGFR was important in all subgroups. Under the assumption of the stochastic process, the latest value is always important to predict the next value. The mean value represents long-term trends, whereas the SD indicates the severity of fluctuation in the short term.

In this study, we employed the widely used Pearson correlation method to identify the top 10 factors with the highest correlation coefficients within each group (Supplementary Table 1). However, these high correlation coefficients appeared to exhibit less variability between groups. This raised concerns about their ability to provide meaningful distinction between the groups. Nevertheless, promising results were obtained through our proposed ML&IVS scheme. We revealed that the crucial factors identified via ML&IVS scheme displayed greater differences between groups than those identified solely through the Pearson correlation method. This finding indicated the effectiveness of our scheme in providing more informative and discriminative features that can potentially improve the understanding and characterization of different groups in this study.

Numerous studies have attempted to identify the risk factors associated with clinical CKD progression (66), and most of them have adopted traditional statistical methods under a theory-driven assumption. In this study, we used ML methods to determine important laboratory factors for short-term eGFR prediction. In all patients with CKD stages 3–5, the latest and mean phosphate levels, SBP variation, latest albumin levels, hemoglobin variation, and mean uric acid levels were important factors in addition to the indicators of current kidney function (eGFR and BUN). Such findings were consistent with those of previous studies reporting that serum phosphate (67), blood pressure (68), lower serum albumin (69), hemoglobin (70), and uric acid (71) levels were associated with CKD progression.

DM is the leading cause of CKD, and approximately 20–30% of patients with type 2 DM have moderate-to-severe CKD (eGFR of <60 mL/min/1.73 m^2^). Such kidney damage is associated with the accumulation of uremic toxins, inflammatory factors, and oxidative stress (72). Therefore, patients with DM have different risk profiles of CKD progression compared with those without DM (73); moreover, proteinuria is usually observed in patients with DM (74) and is well-known to be robustly associated with CKD progression (75). However, in our study, proteinuria was an important factor only in patients with moderate CKD without DM and those with advanced CKD with DM. Such discrepancy may be due to our prediction of short-term eGFR changes. The serum albumin level might be an alternative to proteinuria severity for predicting CKD stage 3 with DM.

The role of uric acid in CKD progression remains controversial. Our results revealed that serum uric acid is an important factor for predicting CKD stage 3 with/without DM. High uric acid levels may cause glomerular injury, tubulointerstitial fibrosis, atherosclerosis, and vascular injuries (76). Some observational studies have revealed that serum uric acid level is associated with renal function impairment (77–79). Moreover, a recent study reported that even uric acid levels under therapeutic criteria may increase the risk of CKD stage transition (74). However, three randomized controlled trials have reported that lowering uric acid levels is not beneficial in preserving renal function (80–82). This discrepancy is attributed to hyperuricemia resulting from renal function impairment. Therefore, asymptotic hyperuricemia does not require medical treatment but needs lifestyle modification.

Phosphorus is an important mineral for maintaining cell structure and energy. It is mainly found intracellularly (70%). Approximately 29% of phosphorus resides in the bones and <1% circulates in the serum (83). In CKD, kidneys cannot excrete phosphorus, resulting in hyperphosphatemia and consequent renal osteodystrophy (84), which was more significant in advanced CKD. A meta-analysis reported an independent association between serum phosphorus level and kidney failure in patients with nondialysis-dependent CKD (67), revealing that higher phosphorus levels might lead to a steeper decline in renal function. Moreover, fibroblast growth factor 23 (FGF23) was secreted by osteocytes in bones, and its levels increased at an early stage (starting at an eGFR of <90 mL/min/1.73 m^2^). FGF23 participates in serum phosphate hemostasis by decreasing phosphorus absorption from the alimentary tract (85). Therefore, hyperphosphatemia may increase FGF23 levels, leading to anemia, cardiovascular disease, and eventually death (86, 87), resulting in a poor renal progression.

This study has some limitations. The dataset used in this study was from a single medical center, which may limit the generalizability of our findings. Therefore, federated learning, which refers to collaborative ML without centralized training data, is necessary to include more data from multiple centers in future studies (88). In addition, the prescribed medications were not collected. Some medicines may have affected the renal function progression. Finally, this cohort consisted of approximately 700 patients, which might have affected the model performance. Next, this study has some advantages. This study used longitudinal data to predict eGFR changes, which can provide a causal inference. The proposed ML&IVS scheme, which integrated the results of risk factor identification and information from five well-known ML methods, could provide more robust and useful information to support our results.

## 5. Conclusion

When longitudinal data are used to predict eGFR changes in patients with CKD with or without DM, the proposed ML&IVS scheme can effectively integrate the most significant risk factors from each model, resulting in more robust and comprehensive identification of important risk factors for predicting eGFR changes. It is crucial to increase awareness regarding eGFR changes, particularly in government health-promotion initiatives within the multidisciplinary CKD care program. This study provides valuable insights for initiating further discussions and follow-up research in this field. These findings contribute to our understanding of the factors influencing eGFR changes and can guide future investigations on the early identification and management of CKD progression.

## Data availability statement

The data analyzed in this study is subject to the following licenses/restrictions: data are available on request to the first author due to privacy/ethical restrictions. Requests to access these datasets should be directed to M-HT, chaomsmyth.tw@gmail.com.

## Author contributions

M-HT: conceptualization, data curation, formal analysis, writing—review and editing, funding acquisition, and supervision. M-JJ and T-CL: formal analysis, methodology, software, and writing original draft. Y-WF: data curation and writing original draft. C-JL: conceptualization, formal analysis, methodology, writing—review and editing, funding acquisition, and supervision. All authors have contributed to the article and approved the submitted version.
