# Supplementary material for: An integrated machine learning predictive scheme for longitudinal laboratory data to evaluate the factors determining renal function changes in patients with different chronic kidney disease stages
Source: Front Med (Lausanne). 2023 Oct 4;10:1155426. doi: 10.3389/fmed.2023.1155426 (PMC10582636; doi:10.3389/fmed.2023.1155426)
Supplement: Supplementary file 1 [file Data_Sheet_1.docx]

*Supplementary Material*

**14. Supplemental table**

**Table S1.** Top 10 Pearson correlation scores in different subgroups

| Rank | **Group 1** | | **Group 2** | | **Group 3** | | **Group 4** | | **Group 5** | |
| --- | --- | --- | --- | --- | --- | --- | --- | --- | --- | --- |
| 1 | eGFR(C) | 0.7 | eGFR(M) | 0.744 | eGFR(C) | 0.853 | eGFR(C) | 0.889 | eGFR(C) | 0.914 |
| 2 | eGFR(M) | 0.659 | eGFR(C) | 0.722 | eGFR(M) | 0.834 | eGFR(M) | 0.849 | eGFR(M) | 0.91 |
| 3 | BUN(C) | −0.338 | BUN(M) | −0.466 | BUN(C) | −0.706 | BUN(C) | −0.682 | BUN(M) | −0.733 |
| 4 | BUN(M) | −0.309 | BUN(C) | −0.434 | BUN(M) | −0.694 | BUN(M) | −0.67 | BUN(C) | −0.73 |
| 5 | eGFR(S) | 0.291 | eGFR(S) | 0.267 | P(M) | −0.595 | P(C) | −0.623 | P(M) | −0.52 |
| 6 | Hb(C) | 0.216 | K(C) | −0.241 | P(C) | −0.537 | P(M) | −0.607 | P(C) | −0.496 |
| 7 | P(M) | −0.208 | Hb(C) | 0.236 | BUN(S) | −0.347 | P(S) | −0.423 | Hb(C) | 0.471 |
| 8 | Hb(M) | 0.205 | K(M) | −0.231 | P(S) | −0.303 | BUN(S) | −0.379 | Hb(M) | 0.455 |
| 9 | P(C) | −0.187 | Hb(M) | 0.225 | Hb(M) | 0.301 | UPCR(M) | −0.349 | UPCR(M) | −0.438 |
| 10 | K(C) | −0.186 | DBP(C) | 0.157 | Hb(C) | 0.297 | Hb(M) | 0.346 | UPCR(C) | −0.431 |

**15. Supplemental figures**

| 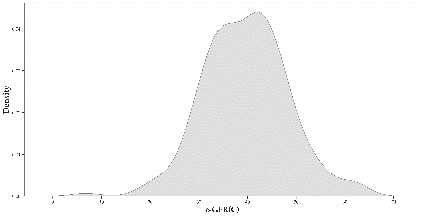 | 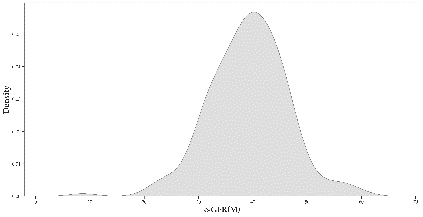 | 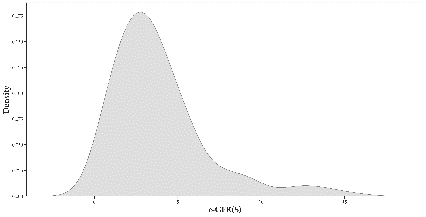 |
| --- | --- | --- |
| (a) eGFR(C) | (b) eGFR(M) | (c) eGFR(S) |
| 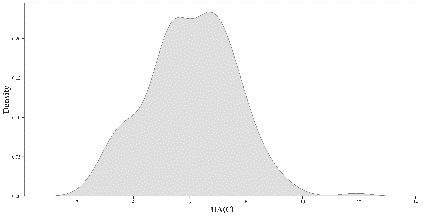 | 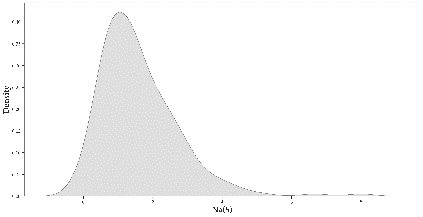 | 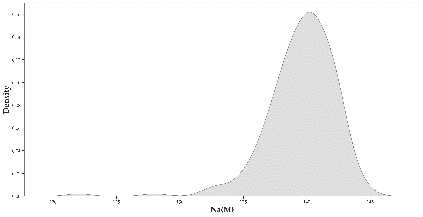 |
| (d) UA(C) | (e) Na(S) | (f) Na(M) |
| 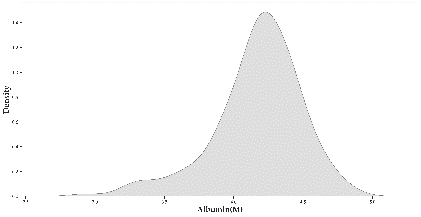 | 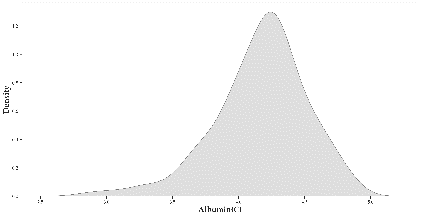 | 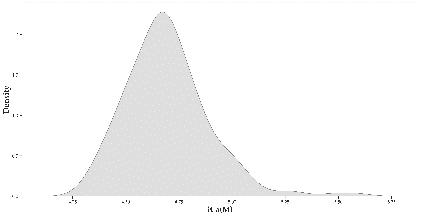 |
| (g) Albumin(M) | (h) Albumin(C) | (i) iCa(M) |
| 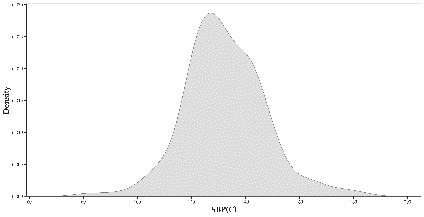 |  | |
| (j) SBP(C) |  |  |

**Figure S1**. Distribution plot of the top 10 important risk factors in Group 1

| 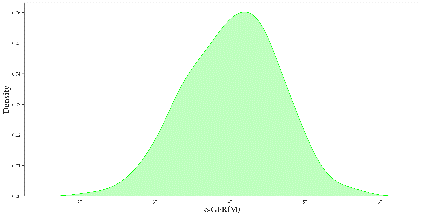 | 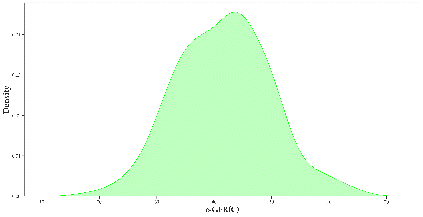 | 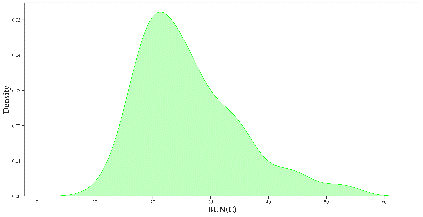 |
| --- | --- | --- |
| (a) eGFR(M) | (b) eGFR(C) | (c) BUN(C) |
| 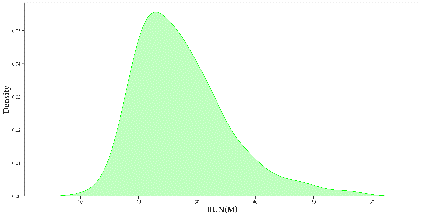 | 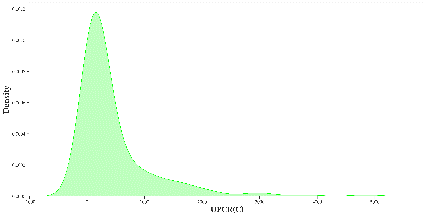 | 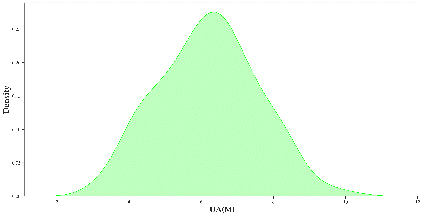 |
| (d) BUN(M) | (e) UP(C) | (f) UA(M) |
| 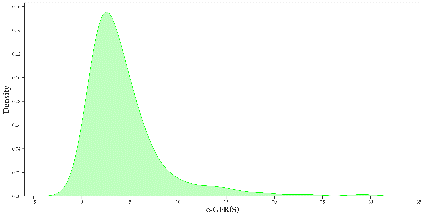 | 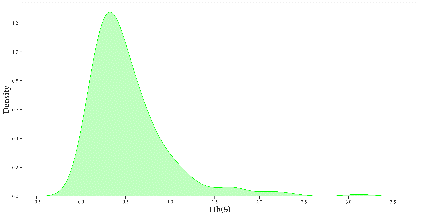 | 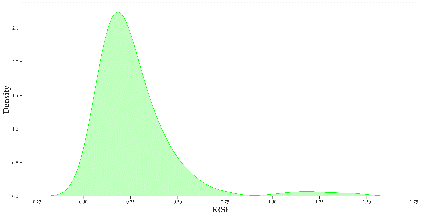 |
| (g) eGFR(S) | (h) HB(S) | (i) K(S) |
| 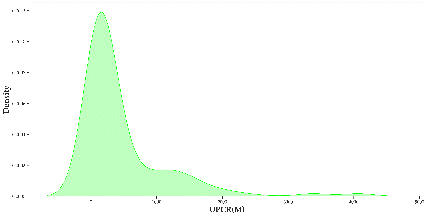 |  | |
| (j) UP(M) |  |  |

**Figure S2.** Distribution plot of the top 10 important risk factors in Group 2

| 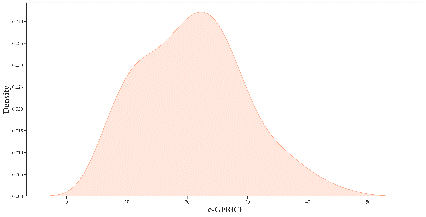 | 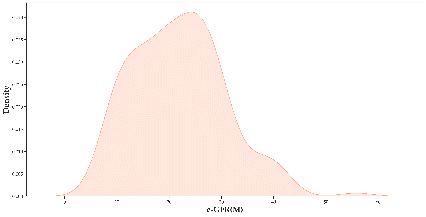 | 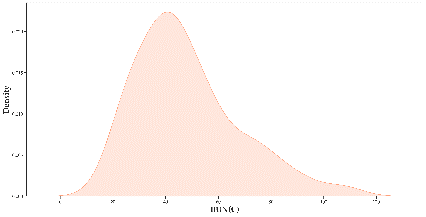 |
| --- | --- | --- |
| (a) eGFR(C) | (b) eGFR(M) | (c) BUN(C) |
| 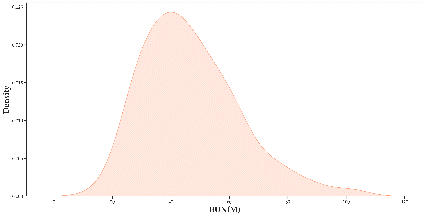 | 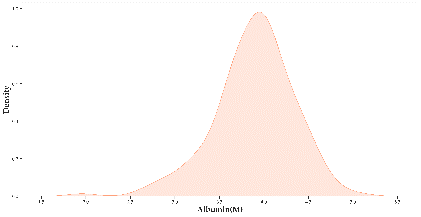 | 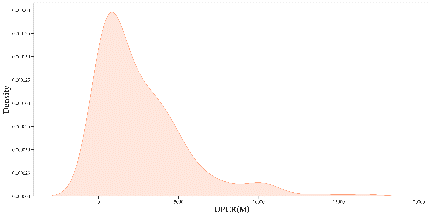 |
| (d) BUN(M) | (e) Albumin(M) | (f) UP(M) |
| 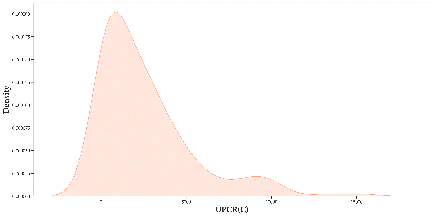 | 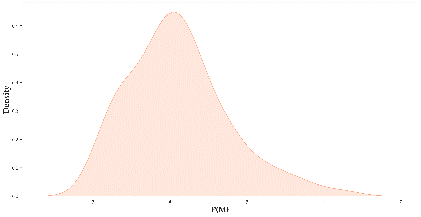 | 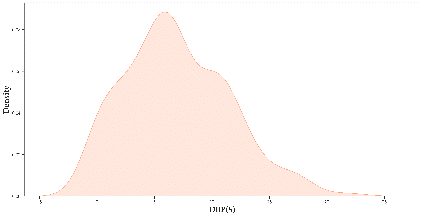 |
| (g) UP(C) | (h) P(M) | (i) DBP(S) |
| 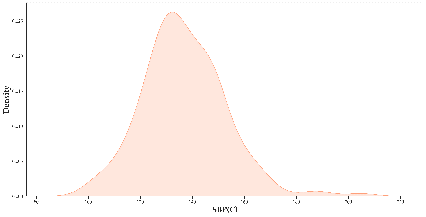 |  | |
| (j) SBP(C) |  |  |

**Figure S3.** Distribution plot of the top 10 important risk factors in Group 3

| 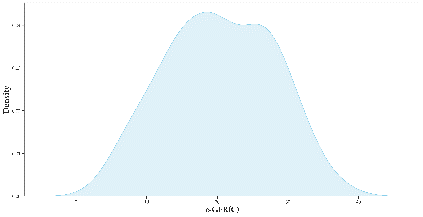 | 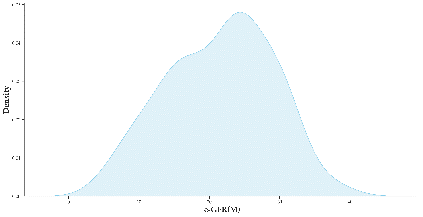 | 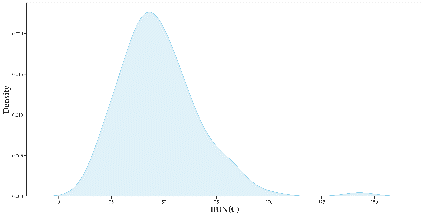 |
| --- | --- | --- |
| (a) eGFR(C) | (b) eGFR(M) | (c) BUN(C) |
| 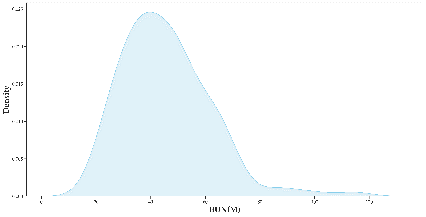 | 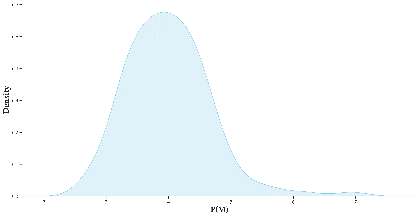 | 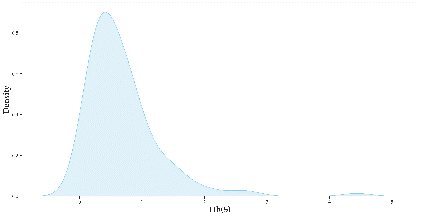 |
| (d) BUN(M) | (e) P(M) | (f) HB(S) |
| 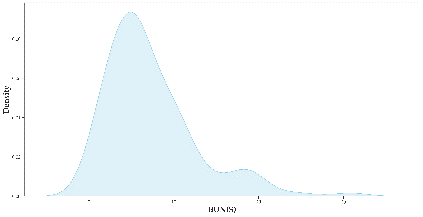 | 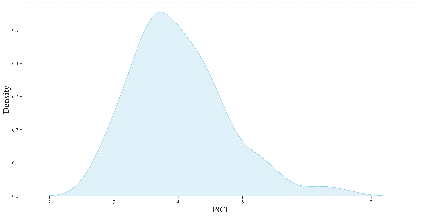 | 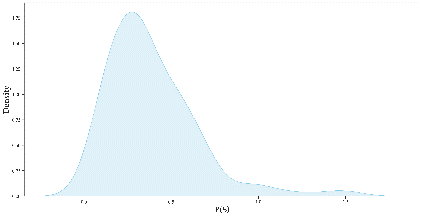 |
| (g) BUN(S) | (h) P(C) | (i) P(S) |
| 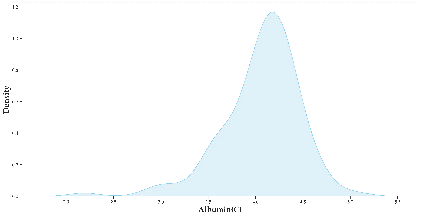 |  | |
| (j) Albumin(C) |  |  |

**Figure S4.** Distribution plot of the top 10 important risk factors in Group 4

| 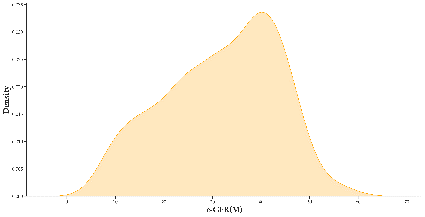 | 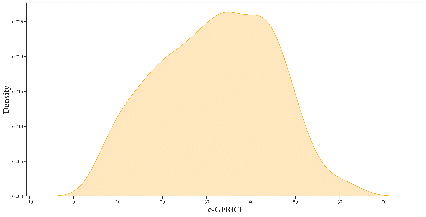 | 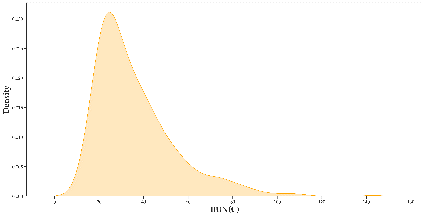 |
| --- | --- | --- |
| (a) eGFR(M) | (b) eGFR(C) | (c) BUN(C) |
| 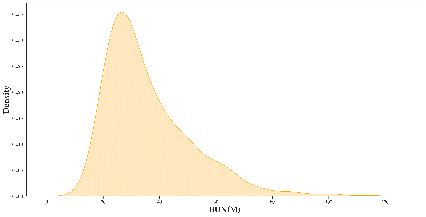 | 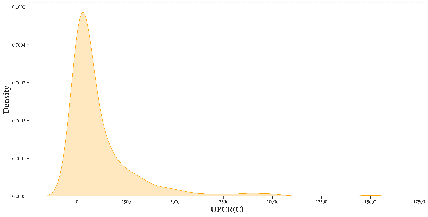 | 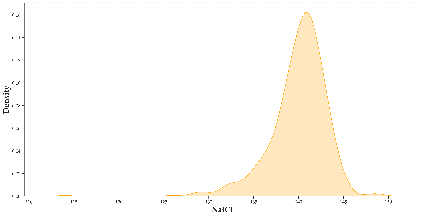 |
| (d) BUN(M) | (e) UP(C) | (f) Na(C) |
| 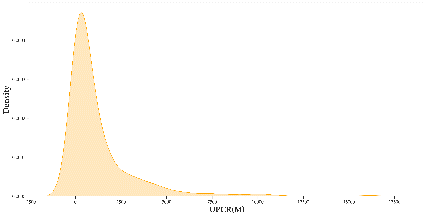 | 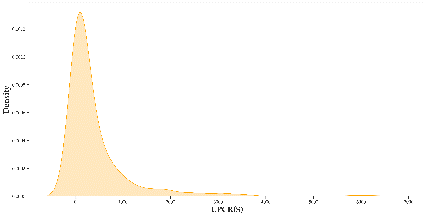 | 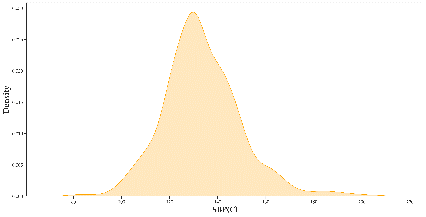 |
| (g) UP(M) | (h) UP(S) | (i) SBP(C) |
| 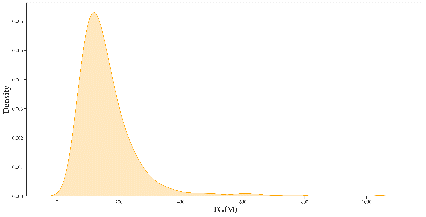 |  | |
| (j) TG(M) |  |  |

**Figure S5.** Distribution plot of the top 10 important risk factors in Group 5
